# What might affect acceptability of online positive psychology interventions for depression: a qualitative study on patient expectations’

**DOI:** 10.1186/s12888-018-1812-x

**Published:** 2018-07-27

**Authors:** Sophie Walsh, Justina Kaselionyte, Stephanie J. C. Taylor, Stefan Priebe

**Affiliations:** 1 Queen Mary University of London, Unit for Social and Community Psychiatry (WHO Collaborating Centre for Mental Health Services Development), Glen Road, London, Newham E13 8SP UK; 2 Centre for Primary Care and Public Health, Blizard Institute Barts and The London School of Medicine and Dentistry, Queen Mary University of London, London, UK

**Keywords:** Positive psychology, Depression, Anxiety, Qualitative, Online intervention

## Abstract

**Background:**

Positive psychology interventions are brief self-adminstered exercises designed to promote positive emotions, behaviours, or thoughts. They are potentially effective for reducing depression and are considered suitable for online dissemination to people with depression and related conditions, as they are assumed to be more acceptable than traditional symptom-focused approaches. However, there is little investigation into perceived acceptability and potential factors that might affect it. This might limit the development and evaluation of effective interventions.

**Methods:**

Semi-structured interviews with patients with depression and/or anxiety (*n* = 18) and professionals, including GPs and psychologists (*n* = 5) were conducted on their perceptions of a proposed online intervention using positive psychology. Thematic analysis, according to Braun and Clarke, was used to identify meaningful patterns in the data.

**Results:**

Four key themes were identified. The fit between the positive psychological approach and the patient’s context, including their personality, symptoms and other treatments, was important in determining acceptability. Social aspects of interventions were thought to facilitate acceptability, as long as these were balanced. Support was identified as important in facilitating intervention suitability, although it was not without limitations. Finally, participants identified how design features can enhance acceptability.

**Conclusions:**

The findings suggest that positive psychology interventions might not be acceptable to all and that specific exercises might be more or less appropriate to deliver online. Design aspects can help to facilitate acceptability, beyond the psychological content. These findings may inform the design of future online psychology interventions for people with depression and anxiety, which can then be evaluated in future research.

**Electronic supplementary material:**

The online version of this article (10.1186/s12888-018-1812-x) contains supplementary material, which is available to authorized users.

## Background

Positive psychology interventions, often delivered as self-help, are brief cognitive and behavioural exercises aiming to increase positive feelings, behaviours, and thoughts that can improve wellbeing and reduce symptoms of depression [1, 2]. Increasingly, individual techniques are packaged together as multicomponent interventions and disseminated to people with clinical and subclinical depression online via websites [3, 4] and smartphone applications (apps) [5]. Such online dissemination is a strategy to sustainably improve access to mental health interventions [3, 6] given the vast numbers of people globally experiencing depression, and related common mental disorders such as anxiety [7].

In order to develop such interventions and successfully evaluate whether they are effective, the Medical Research Council (MRC) recommends first testing and refining the intervention to ensure it is acceptable [8]. Acceptability can be defined as the extent to which an intervention is suitable, satisfying, and attractive, to potential users [9]. Further, when developing an online intervention, which patients often use independently, it is particularly important to elicit, understand, and accommodate patients views to reduce psychological and practical barriers and to maximise acceptability [10].

Acceptability has been explored within the literature on computerised interventions, which are traditionally informed by CBT [11]. For example, a recent qualitative meta-synthesis of patient experiences of computerised CBT or related treatment indicated that acceptability may depend on how sensitive the computerised intervention could be to individual needs and how much collaborative support it provided [12]. However, to date there is no clear literature on the acceptability of positive psychology interventions to people with depression. Positive psychologists have expressed favourable expectations that interventions might be more appealing and have fewer barriers to entry for people lacking motivation, energy, or enthusiasm, when compared to accessing traditional forms of therapy such as CBT or other deficit-oriented approaches which focus on ameliorating a presumed deficit or problem [4, 13, 14]. It is proposed that the resource-oriented nature of positive psychology interventions is appealing, i.e. the focus on the internal resources such as strengths and external resources such as friends and family to promote therapeutic change [15]. Put simply, it might be more appealing to complete an inventory of strengths rather than an inventory of symptoms. Anecdotally interventions are reported to generate overwhelmingly positive feedback even with patients with clinical depression [14].

However, few studies have provided data to support the idea that positive psychology interventions are acceptable. One study reported that almost 60% of participants with depression were indifferent to, or dissatisfied with, an online intervention using components of positive psychology however, did not collect data on reasons for dissatisfaction [3]. These researchers suggested that participants might have been dissatisfied with the intervention content, and felt unable to complete it, or that the intervention may have lacked suitably attractive design. Others have suggested that people with depression may find positive psychology interventions inappropriate or unattractive [16] as, by its nature, depression is associated with reduced interest in previously enjoyable activities and deficits in motivation [17].

The main focus of positive psychology research has been investigating the factors that could influence intervention effectiveness. For instance, Lyubomirsky and Layous [18] propose that the success of a positive psychological intervention may depend on the characteristics of the intervention (e.g. social support) and the characteristics of the person (e.g. their affective state), both of which can influence each other to determine an optimal person-activity fit. Thus far the evidence for how depression and related disorders influences intervention outcomes is reported to be inconclusive [18]. However, it has been suggested that acceptability can influence outcomes, as patients who perceive interventions to be suitable and attractive appear more likely to benefit [19, 20].

Further research is needed to investigate the acceptability of positive psychology interventions to people with depression as the lack of conclusive data limits the ability to design potentially acceptable interventions for this population. Many patients with depression also meet the criteria for another psychiatritc condition, often anxiety (Kessler et al., 2003) and to ensure that the present study generates results that are relevant to clinical practice, participants with depression and/or anxiety will be included.

One way to explore the potential acceptability, needs, and preferences of the intended audience is by employing qualitative methods which are perceived as particularly valuable in the design and development of online interventions for health [10]. However, to our knowledge, no studies have yet qualitatively investigated perceived acceptability of using positive psychology online.

The aim of this study was to explore patient and healthcare practitioner views of a proposed online intervention using positive psychology to address the research question: What is the acceptability of an online positive psychology intervention for depression and anxiety? The findings may help to inform the design of future online positive psychology interventions for people experiencing common mental health disorders and increase the acceptability of such interventions.

## Methods

As the study aimed to understand personal attitudes an in-depth semi-structured interview was deemed most suitable. This method allows discussion of areas the researcher wishes to cover, according to a topic guide, whilst giving the interviewees the opportunity to explore their different thoughts and feelings [21]. Thematic analysis is an appropriate method to identify salient and recurrent themes [22]. The conduct and reporting of the current study adheres to the consolidated criteria for reporting qualitative research (COREQ) [23]. Local research governance and national ethics approvals were received for the study (North West - Preston National Research Ethics Committee 15/NW/0349).

### Sampling

Invitations for participants were placed in the United Kingdom (UK) National Health Service (NHS) and community treatment settings across East London which included General Practices, counselling services and charities. Online adverts were placed on Twitter and Facebook. We sought participants willing to discuss the design of a future online positive psychological intervention. As depression often occurs alongside anxiety [24] the present study included participants if they reported accessing treatment for their depression and/or anxiety in the previous 12 months and were aged 18–65. Participants could not participate if they had insufficient command of English, or had a diagnosis of Bipolar Disorder, as there is no evidence yet on the suitability of positive psychological interventions for this population. Inclusion and exclusion criterion were assessed on the basis of participant self report, via brief phonecall prior to the interview. Healthcare professionals were invited to participate if they had at least 12 months experience of clinical responsibility for managing the depression and/or anxiety of working age adults. Primarily, we used purposive sampling, with some snowballing techniques, paying attention to participants’ age, gender and treatment setting to ensure we captured a range of experiences. It is typical in qualitative research for sampling strategies to overlap, given the flexible, pragmatic nature of qualitative enquiry [25]. Participants were selected for interview until data saturation was achieved, i.e. the authors felt that additional interviews did not provide new ideas.

### Study setting

Participants were required to provide written informed consent and then complete a short demographic questionnaire detailing age, gender, first language and personal experience of website and app use both in daily life and to manage their health. The interviews were based on a semi-structured topic guide (provided in Additional file 1) that included key questions and suggested probes. The topic guide was developed with the study team and piloted and iteratively refined following early interviews. Participants were asked their views on the homework exercises from individual positive psychotherapy [14], summarised in Table 1, as this is the best-described package of positive psychology interventions. Participants were shown Table 1 and the interviewer described the exercises but no other prompts were used. Participants were also asked views on the structure, features and design of an online positive psychological intervention the mode of which was not specified as a website or app.

**Table 1:** Positive psychology homework exercises from individual positive psychotherapy

Exercise	Brief description
Positive Introduction	Write short story at your ‘best’
Identifying strengths	Person completes Values in Action Inventory of strengths (VIA-IS). Their family/ friend completes shorter version
Strengths plan	Plan to use top strengths in daily life
Blessings journal	Record three good things per day
Writing memories	Write three bad memories and distress
Forgiveness letter	Write to someone that want to forgive but do not deliver letter
Gratitude letter	Write to someone never properly thanked and deliver letter
Personal satisficing plan	Plan to settle for good enough compared to trying to find the ‘best’ option
One door closes, one door opens	Write times when something important did not happen but other opportunities did
ACR	Practice being enthusiastic and supportive of others’ meaningful and important news
Family strengths tree	Each family member completes VIA-IS followed by group discussion
Savoring activity	Attempt to make pleasure last
Gift of time	Use strengths in the service of others

Interviews were conducted by SW in a private research office and lasted between 34 and 130 min, with an average length of one hour. Patients received £20 remuneration for their participation.

### Analysis

Interviews were audio recorded, transcribed verbatim and potentially identifying details were removed to ensure anonymity. The researchers (SW and JK) approached the data with a realist viewpoint, whereby participants accounts are seen as grounded in reality, whilst acknowledging the role of social context [26]. The data were analysed inductively, and codes were created that were specifically relevant to the research question using thematic analysis [22]. The analysis process was iterative and early analysis informed subsequent interviews. Following initial familiarisation the first researcher (SW) coded all transcripts, applying brief phrases or ‘codes’, to summarise pertinent phrases or sentences using NVivo software. All transcripts were analysed concurrently to establish themes across participants, rather than to compare participants with different characteristic (such as patients vs. clinicians or those with different diagnoses). This approach was in line with the aim of the study to broadly understand the acceptability of positive psychology, to inform the design of future online positive psychology interventions for people experiencing common mental health disorders.

A second researcher (JK) conducted credibility checks on 20% of coded interviews to ensure the consistency and credibility of coding. The codes were sorted into preliminary themes and sub-themes that were repeatedly discussed by the authors and a patient advisory panel linked to the wider project. The panel consisted of four patients with lived experience of anxiety and depression who responsed to adverts placed within local services and patient participation networks. Members were selected on the basis of demonstrating interest in the project and having relevant experience. Guidelines for establishing reliability and validity in qualitative research were used, with a focus on ensuring the coherence, distinctness and credibility of themes and sub-themes [27]. When agreement was reached on the themes the researchers (SW and JK) worked together to ensure the analytic narrative represented the data, in relation to the research questions, which is presented in the subsequent results section alongside interview excerpts. The credentials and possible influences of the research team members on the study conduct and analysis are provided in Table 2.

**Table 2:** Research team and reflexivity

	Author 1 SW	Author 2 JK	Author 3 ST	Author 4SP
Professional role and credentials	Health services researcher, BSc	Health services researcher, MSc	Health services researcher and GP	Psychiatrist, psychotherapist, researcher
Role in the research	Interviewer, lead analyst	Support data analysis	PhD supervisor	PhD supervisor
Potential influence on interview conduct or analysis	Established relationships with interviewees Lead on project developing an online positive psychological intervention (PhD project) Familiarity with positive psychology and eHealth literature	Familiarity with mental health services research literature	Familiarity with eHealth literature	Familiarity with resource-oriented treatments and existing mental health service practice and literature

## Results

### Participants

Eighteen patients participated. They were predominantly female (78%), spoke English as their first language (89%), and were on average 38 years of age (range 20–65). Most patients reported receiving treatment for their depression (61%), with some receiving treatment for depression and anxiety (22%), or primarily for anxiety (17%). Five healthcare professionals participated, including two general practitioners, two low-intensity psychological therapists and a clinical psychologist. The clinicians in the study were predominantly male (60%), generally spoke English as their first language (60%) and were on average 37 years of age (range 30–49). In the whole sample, nearly all participants reported smartphone ownership (96%), with the majority reporting using apps daily (87%), although fewer people reported using apps (30%) or websites (43%) for managing their health.

### Themes

The following themes were identified in relation to the research question: ‘The fit between a positive psychological approach and context’, ‘Balancing the social’, ‘The role of support’ and ‘Persuasive design’. An overview of the themes and related sub-themes is provided in Table 3.

**Table 3:** Themes and subthemes

1. The fit between a positive psychological approach and context	2. Balancing the social	3. The role of support	4. Persuasive design
1.1. Ability to identify positives 1.2. Feeling misunderstood 1.3. A complementary approach 1.4. Cultural fit	2.1. Connecting to overcome self-absorption 2.2. Complexities in social relationships 2.3. Technology: promoting isolation or connection?	3.1. Managing emotions 3.2. Promoting independence vs. motivation 3.3. Service capacity	4.1. Appeal and accessibility 4.2. How much is expected 4.3. Credibility 4.4. Tailoring and targeting

#### The fit between a positive psychological approach and context

A key factor in engagement was how well a positive psychological approach fitted with the patient’s context such as their personal ability to find positives, their feelings towards the approach, their current treatment and their culture.

##### Ability to identify positives

Participants reported that they may not have the ability to find positives and this might affect them starting the intervention. Participants identified a few factors that may influence this ability. Firstly, personality was thought to be important. If positivity is not a natural fit with someone’s disposition or outlook on life, they may have difficulty engaging with this intervention.

‘This sounds really awful but people that are negative in general, even when they are well, tend to have quite a negative outlook on things – those are the people that are at more of a risk I think of not responding well to this treatment because their general…some people’s personality is negative.’ (Patient, 0504).

A second factor is the nature of depression itself, where the default negative thinking mode may limit one’s ability to identify positives. Patients described a tendency to ‘filter out’ positives as they seem irrelevant and incongruent to their affective state.

‘When you’re down automatically everything comes out negative. You’re never gonna say alright, I’m negative and I want to think about positive things because the mind won’t let it.’ (Patient, 0401).

The final factor is that the social circumstances of people experiencing depression or anxiety may not always be positive, which limits their ability to find positive moments.

That probably was the lowest point in my life, I think I probably would have found it really hard, ‘cos actually things weren’t going well at that time – actually […] I hadn’t found a job, relationship was in pieces, like things were pretty strained with my parents […] so I think it would be really hard [laughter] to like, like drawing blood out of stone… (Patient, 0502).

##### Feeling misunderstood

In the context of depression and anxiety, participants felt that being offered a positive psychological intervention could result in them feeling dismissed, belittled and misunderstood and thus unwilling to use it. Participants likened the approach to common unhelpful responses to depression, such as ‘be happy’ or ‘what have you got to feel depressed about?’

‘I think the biggest kick in the teeth when you’re feeling particularly depressed is when it almost feels like people are putting your feelings to one side and saying oh, shh, you know just stop it, whatever, feel good’, (Patient, 0503).

‘I mean that’s…I think when you’re suffering through a dark time, even the phrase positive thinking is enough to make you sick; it seems like you know a phrase with which people are refusing to understand you,’ (Patient, 0901).

These figurative expressions ‘kick in the teeth’ and ‘make you sick’ indicate the frustration that can result if patients feel their problems are being reframed as less serious, or as not worth exploring. Patients indicated they might not be willing to try an approach that drives a notion of positivity without validating their feelings as real and painful.

Participants expressed that an intervention should be suited to their context and allow exploration of ‘negatives’.

I know it’s all about positivity, which is fantastic – we all need positive vibes […] but I’m wondering if […] you can look at where you don’t wanna go […]. So when you’re feeling really bad you know like self-medicating, self-harm, that’s where I don’t wanna go. (Patient, 0702).

This indicates that a more rounded approach, which patients feel is relevant to their context, may be more useful than a solely positive approach.

##### A complementary approach

Participants discussed there may be issues with engaging with a positive psychological intervention unless the patient’s treatment context includes approaches that address their symptoms and problems. To enable patients to meaningfully engage, participants described that a positive psychological approach might need to be introduced alongside other treatments such as medication that can manage symptoms, or a solution-focused therapy to address problems.

‘If […] you’ve been, you know really depressed for six months or something like that, then you’d probably have to be medicated in order for you to kind of have the presence of mind in order to focus on the positive aspects.’ (Patient, 0506).

This led to a discussion of when to introduce the approach to best engage people. Some felt you first need to let people recognise their symptoms and deal with them. Yet, it was acknowledged that you might miss the chance to intervene and there is a benefit to introducing a positive psychological approach early on.

‘It might not be easy for them to do it immediately, but it’ll certainly make them think oh well this is stuff I can work towards and do and be positive about enjoying the positive emotions that come with it,’ (Psychological therapist, 1402).

Overall, participants highlighted that an online positive psychological approach might engage people when it is offered as a complementary approach.

##### Cultural fit

Another important aspect of the fit between a positive psychological approach and context that could affect patient engagement, relates the use of an American approach in the UK. Whilst the pursuit of happiness is enshrined in the American constitution, it was not seen as the British ‘way’.

‘A lot of the American terminology is […] construed by British people to be a bit over the top.’ (Clinical Psychologist, 1403).

The sense was that positivity might not be valued, or taken seriously, in the UK. Participants associated many of the terms and activities with religion, such as ‘forgiveness letter’, ‘blessings journal’ and ‘gratitude letter’, which may not suit everyone.

‘You just need to word it differently and yeah. ‘Cos it sounds a bit kind of churchy and yeah, like happy-clappy yeah[h]. […] it needs to be a completely non-religious thing that you’re gonna do […] you want people of all different faiths and people that haven’t got faiths to be able to do it’(Patient, 0504).

Further, whilst gratitude is exemplified in North America’s national holiday, Thanksgiving, it was perceived differently in the UK:

‘I don’t even like the word ‘grateful’ because I’m like, well I am very grateful but I don’t really need to be told to be grateful unless you’re being like a spoilt brat – that’s different. So I’m just a bit like wary of that word because […] [its] very like ‘this is the way’ like gratitude, like that’s what you should be feeling’ (Patient, 0903).

This view suggests that some of the language is likely to be a factor in engagement with an online positive psychological intervention in the UK.

#### Balancing the social

The extent to which social aspects of positive psychological interventions were balanced was an important factor in patient engagement.

##### Connecting to overcome self-absorption

Participants recognised the need to connect to overcome self-absorption, i.e. caring only about their difficulties and isolating themselves. They identified that social positive psychological exercises, such as the ‘gratitude letter’, in which participants thank someone they have never properly thanked, and ‘active constructive responding’ in which people learn to respond favourably to others’ good news, could help. Benefits include identifying your support network, spreading positive news amongst this network and strengthening social bonds. Similarly, participants discussed the benefits of connecting with the wider community, through the ‘gift of time’ activity, in which they are invited to use their personal strengths in the service of their community.

‘To make myself feel better like I do volunteer […] it’s just like a good thing to do to like get out of yourself, and to go do and then like come home and you’ve been out of the house but it’s for a purpose.’ (Patient, 0903).

This quote illustrates how connecting can raise awareness of others’ needs, improve mood, develop a sense of purpose and get you out of the house. These benefits can be important factors in patients starting a positive psychological intervention to redress imbalance in their social lives.

##### Complexities in social relationships

Despite possible benefits, participants felt that a barrier to benefitting from social aspects of the intervention could be complexities in social relationships and interactions that are difficult to navigate when unwell.

‘I might be thinking very positive myself, I may not come out like that. You know so if it comes out like you know very sort of [pause] blurry and[…], slow or it doesn’t come out – my facial actions, my eye contact, stuff like that – if it’s not connecting then maybe words doesn’t make no difference’ (Patient, 0703).

Participants also discussed issues with taking on others’ needs when you have your own. For example, using your strengths in the service of others in the ‘gift of time’ activity.

‘The only thing that I am a bit hesitant or sceptical about is about the voluntary side of stuff – especially if you can’t give too much time or effort because actually maybe you’re actually in need of things yourself’ (Patient, 0902).

Participants discussed which social relationships should be utilised, and felt that activities involving family members (e.g. ‘family identifying strengths’ in which family members complete a strengths inventory about the person, and ‘family strengths tree’ in which family members complete their own strengths inventory and compare results amongst family members) were less likely to be started. This is due to complex dynamics and potential for conflict. It was suggested that involving a broader social network should be promoted.

‘Something that’s…that encourages an open, alternative form of relationship – it’s about fostering relationships. So …and family is just one of those. And now you can have a family of friends, you know?’ (GP, 1401).

Another way to balance the benefits of involving social contacts is to avoid activities that may lead to a direct discussion of mental state (e.g. ‘family identifying strengths’, ‘family strengths tree’). Participants described that their mental health is private, is often misunderstood by others and discussions may be too difficult.

This sub-theme illustrates the importance of allowing patients to choose the most appropriate activities, with their preferred social contacts, in order to balance the benefits of social activities with their potential demands.

##### Technology: Promoting isolation or connection?

Whether people perceived technology as having the potential to promote isolation or social connection was a factor affecting their likelihood of using, or recommending, an online positive psychological intervention. For some, technology was viewed as inherently isolating as it is separate from the social world and may therefore be unsuited to those already isolated due to their mental health condition. Despite recognition that technology can enable access to treatment without leaving the house, this was also viewed as reducing opportunities to meet others, to discuss feelings, and to take advantage of encounters in the social world.

‘Where depression may relate to isolation, I think technology is inherently isolating, so I think you’d probably meet some resistance on that front’ (Patient, 0901).

Counter to this view, others felt that when balanced, i.e. used as a complementary tool, technology has the potential to promote awareness of their social networks and social connectedness.

‘We’re not saying it’s the be-all and end-all, but definitely has a place to get people understanding information or you know ideas. And it…one of the ideas could be this outside-in approach that’s on the app, so they know that it’s not just about the phone, it’s about doing things for friends and family’ (Psychological therapist, 1402).

Participants preferred a tool that promoted real-world connections, e.g. mentioning the person’s family or friends and including a directory of local community services, rather than online social networking features such as a forum.

‘If you want to speak to other people that are going through something similar, it’d be good to have that option but then I suppose on the negative side of that do you really want lots of negative people talking to other negative people, because it could end up putting you in a downward spiral.’ (Patient, 0704).

This quote suggests that despite benefits of online support networks, there are potential risks that undermine the intervention aim. However, participants felt a sense of social connection could be fostered through users sharing positive resources, or progress updates, so that patients feel less ‘alone’ with the online intervention.

#### The role of support

Participants described various ways therapeutic support from a person could function to encourage use of the intervention.

##### Managing emotions

Participants described that a key function of therapeutic support could be to manage emotions associated with their depression and anxiety by allowing people to explore their difficulties in a safe space. The empathy and respect that could be provided was seen as validating, whereas not providing that personal support was viewed as potentially dismissive. Participants expressed concern about managing the emotional fall out from exercises exploring memories or events, such as ‘writing memories’ in which participants write three negative memories, the ‘forgiveness letter’ in which patients write forgivness to someone, or optimism exercises such as ‘one door closes, one door opens’ in which participants recall how an apparently missed opportunity led to another event.

‘How would the app say to you oh […] it wasn’t your fault, or this shouldn’t have happened, or it’s not that bad, or like it has happened but you’ve gotta put it in the past – an app can’t tell you that. That could be quite, that could be good and it could be very bad; that could trigger something off couldn’t it?’ (Patient, 0101).

This quote highlights the supportive interactions that may be required to explore and process emotional reactions to some activities. Without this support, there was a sense that certain activities may lead to emotional worsening rather than improvement.

##### Promoting independence vs. motivation

On the one hand, participants identified that therapeutic support would enable them to understand how to use the tool, check their progression and receive encouragement. All of this could promote their motivation to use it.

‘Yeah, ‘cos I’m a terrible sort of procrastinator or whatever – I can put anything off, unless other people are involved and then, if you’re accountable then you do it.’ (Patient, 0501).

This view indicates that beyond encouragement, having support creates accountability that can improve engagement. However, there was a tension between promoting engagement and motivation and limiting patient autonomy and development of self-management strategies.

‘You know what if they give up then that’s their choice isn’t it? But I think if you say…and I check this [taps the table repeatedly] and you’d better do it because I will check it, then it’s not self-help anymore’ (GP, 1501).

Evidently, although it may help to provide support and may increase the chances that the intervention will be used, it must be balanced with the aim of the intervention which is to promote autonomy and personal development of positive strategies.

##### Service capacity

Whilst it was recognised that therapeutic support may promote engagement and serve patients’ emotional needs, participants questioned the capacity of services to provide this given the current struggle to provide follow-ups and check-ins.

‘And I think it needs support and I think this is where…. this is what is lacking in GP surgeries and all over the country, is the support. The support network, befriending network – like people like MIND [mental heath charity] who, you know do a fantastic job’ (Patient, 0505).

This highlights that non-statutory services including charities currently provide support. Linked to this, patients indicated it was not necessary for an in-person support but perhaps remote support via phone or email may be useful. However, participants recognised that even if such low-intensity support is provided this still comes with service implications.

#### Persuasive design

Participants identified that numerous features of an online technology could be persuasively designed to optimise the take up and use of the intervention.

##### Appeal and accessibility

For an intervention to be appealing and accessible, participants wanted information presented in an engaging way.

Presented in a […] way where it’s softer round the edges […] so it looks a bit more like an app that you’d wanna kind of play with, but it’s actually helping as well, rather than being something that’s like medical and psychologically necessarily termed. (Psychological therapist, 1404).

This quote highlights how language, layout and interactivity can facilitate engagement. Clear, simple explanations were preferred more than complicated terms. Participants hoped the tool would be interactive and make use of online features, e.g. touch screen technology and multimedia videos or audios. They did not want a tool which simply presented text and asked them to input text. Yet, the tool should be simple to use.

‘Maybe not necessarily how it looks but how you actually use it would be quite important to me. I often notice things in apps that like are a bit ‘buggy’ or actually that’s a bit of a pain to do.’ (Patient, 0201).

Good design therefore includes the design of the interface itself. It also includes how the tool looks. Participants mentioned the importance of staying away from a contrived, traditional ‘self-help’ feel and avoiding using out-dated imagery and graphics, but instead aim for a positive appealing impression to facilitate engagement with the tool.

##### How much is expected

Participants were less likely to engage where an online positive psychological intervention included time-consuming or effortful components. Since positive psychology exercises vary in effort required, participants preferred those needing lower input, including lower emotional effort.

I guess if you’re doing it [forgiveness letter] in the sense of an app I wouldn’t…like things which is like questionnaires and you know write three things positive about yourself and all that, like I think that sounds all good, because it’s like much more surface level, whereas a letter is much more, you know it’s getting to the nitty-gritty. So I probably wouldn’t…yeah I’d probably just skip over. (Patient, 0903).

An important aspect related to expected effort was how the activities were presented in the technology. Participants discussed the relative benefits of presenting all content, compared to a sequential presentation of new content on a fixed schedule.

I think you should have more than one because I think people lose interest quickly if it’s just one thing I think.’ (Patient, 0101).

I wouldn’t wanna be bombarded, because one a day is quite good, one at a time, and then you go on to a next level. Whereas if it’s all at once you don’t know what’s hitting you and you can get bored with that, and you give up, ‘cos it’s too much – overwhelming. (Patient, 0701).

Whilst there are varying preferences on how to present activities to maintain participants’ interest, where possible the priority should be to ensure people do not feel that too much is expected of them.

##### Credibility

Participants discussed that they would be more likely to use an online positive psychological intervention that is credible, i.e. trustworthy and reliable. For some, this meant a GP recommendation, or NHS branding.

It makes sense [to have the NHS logo] because then I think people trust this app more than when it’s just a commercial thing. Yeah, it’s like you know when they look on the Internet for advice, they always trust ‘NHS Choices’ or the NHS website more than any other website. (GP, 1501).

However, it was acknowledged that credibility comes in different ways for different people.

So somebody’s opinion that you trust whether that’s a person in position of authority or a person that you know has been through similar things. (Patient, 0506).

What is important is a trust in the source that recommends the tool. But also, the tool itself needs to appear legitimate and credible, in terms of offering appropriate advice, working reliably and reflecting patient perspectives.

##### Tailoring and targeting

Participants felt that where an intervention was tailored and targeted, this may facilitate independent use of it. For instance, participants recognised that reminders might be useful to help overcome memory and cognitive deficits. However, patients felt that beyond simply prompting people and tailored reminders could be used to incentivise and engage.

I think it’s gonna be hard to rely on somebody to log back in and look at the good things that they’ve said about themselves that day […] so it’d be good to have something to come up and say look, this is what you’ve said about yourself in the week – just to make you feel good. (Patient, 0704).

If reminders are well designed they could provide positive reinforcement to continue, thus making an intervention self-propelling rather than reliant on a third-party for encouragement.

Progress tracking was also seen as persuasive as it allows people to quickly visually check their progress e.g. in the intervention itself, or towards a particular goal, and may promote motivation to continue.

Participants reported they would be more likely to use an intervention that provides practical suggestions on applying the exercises to their lives because it is difficult to come up with ideas. However, there are issues with providing examples.

Cos that was something about ‘Beating the Blues’, that some of the examples are quite specific and they were quite difficult to relate to if you weren’t whatever, 44, you were an old man or whatever it was. (Clinical Psychologist, 1403).

Again, this view hints that personalisation may be useful so that examples could be tailored according to patient demographics (e.g. age, gender, and living situation) and symptoms.

Finally, in order to maintain patients’ interest in the intervention it could release relevant novel content.

You know there’s an algorithm that goes right they’re thinking about that thing so that kind of unlocks a different element about it […] you know if they are engaged in doing this exercise then having loads more unlock levels of that thing I suppose. (Psychological therapist, 1404).

The reward of getting novel content, aligned with patients’ interests, may be a more engaging approach than releasing content in a pre-defined sequence and could facilitate independent use of the technology.

## Discussion

### Summary of key findings

Our findings indicate that numerous factors could affect how acceptable people experiencing depression and anxiety might find an online positive psychology intervention, and what barriers exist that may impede uptake. A critical factor is the fit between the positive psychological approach and the patient’s context, including whether patients feel they can identify positives, whether they feel understood, whether it complements other treatments and whether it culturally fits. A balance is needed between the promotion of positivity and validation of symptoms. Another important factor is social support, which patients felt could be leveraged to encourage use of the intervention. However, this needs to be balanced and flexible to allow patient choice in who to involve and in which activities. This should also promote connections in the social world. Participants identified that therapeutic support that helps to manage emotions might be an important factor in whether someone uses an online positive psychological intervention. However, participants acknowledged the tension between providing support and encouragement, whilst potentially limiting a person’s capacity to autonomously develop positive strategies. A tension between the use of technology being both enabling to connect to others and potentially isolating was also highlighted. Finally, participants identified that a persuasively designed intervention was likely to be acceptable if it was accessible, credible and did not require too much effort.

### Strengths and limitations

To our knowledge this is the first study to explore patient and healthcare professional preferences on an online positive psychological intervention for people with depression and anxiety. The use of a qualitative approach enabled a nuanced understanding of potentially relevant factors affecting acceptability and uptake. This is important to ensure that such interventions are designed in a way that optimises acceptability, based on evidence. Given the widespread interest in taking positive psychology online for clinical and subclinical populations, [3, 5, 6] it is salient to ensure that interventions are not undermined by issues of acceptability [8]. Despite this strength, the study has some limitations. Firstly, participants were self-selected and so may not be representative of the views of those less interested in an online positive psychology intervention. Whilst this is not necessarily a critical limitation, as by its nature online self-help is accessed by self-selected help seekers, it is important to be aware that we perhaps may not have identified all relevant factors in the acceptability of online positive psychology interventions in a sample of motivated participants. Secondly, the researchers’ backgrounds may have influenced the findings as the interviewer and lead analyst (SW) aimed to develop an online positive psychological intervention and all interviewees were aware of this. As a consequence, social desirability bias may have led to under-reporting of possible barriers to accessing such an intervention. However, the analysis was conducted with a multidisciplinary team and excerpts have been provided to support authors’ interpretations. Finally, the interviews were discussing the future design of an online positive psychological intervention and what participants imagine will be important and relevant, which may differ from what is actually important in practice. Despite these limitations the study findings remain relevant, since it is critical to investigate perceptions prior to designing digital tools [10].

### Clinical and research implication of findings

It may be useful to consider the present results in light of Lyubomirsky and Layous’s positive-activity model [18] that indicates the success of a positive psychology intervention can depend on person and activity characterstics that can influence each other to determine an optimal person-activity fit. Thus far the evidence for people with depression and related disorders is reported to be inconclusive [18]. However, findings from the present study indicate further avenues for exploration.

Firstly, the present findings highlight which exercises might be optimal for people with depression and related disorders. The positive-activity model indicated that social activities may be more suitable for those isolated than reflective-cognitive activities [18]. Also, the authors suggested the degree of perceived social support was likely to be relevant. Our findings support both hypotheses, as participants discussed a preference for activities based within social networks, rather than reflecting on personal feelings. Further, participants indicated that social support was relevant, particularly in terms of activities being practiced in the real-world, rather than through online support networks. However, the present findings indicate the importance of balancing social demands, with activities such as the ‘gift of time’ perhaps being less suitable. Future research may consider the specific barriers and benefits of such social activities for people experiencing common mental health disorders.

Secondly, the present research provides some important detail on how well a positive psychological approach fits with people who are depressed or anxious. Previously it has been assumed that people with depression may be relatively more likely to benefit as they may be motivated to find the positives [13]. However, our findings indicate that there are specific barriers for people with depression and anxiety including feeling the inability to find positives and feeling misunderstood by the approach. Whilst positive psychologists have been sensitive to the criticism that the approach fails to account for patients negative emotions [28, 29], it must be acknowledged that the context is perhaps different online. In a therapeutic environment a therapist can validate negative emotions, without exploring them, and focus on prioritizing the positive. This may be more difficult to achieve online, without interaction with a therapist. Further research is required into ways to present the positive approach so that it does not appear dismissive of patients’ symptoms.

Further, our findings indicate that the current treatment context is likely to be an important factor in engagement. Others have suggested that positive psychological interventions may be more useful for people who are not benefiting from or not accessing traditional treatments [13]. However, in the present study patients indicated they might be more likely to select and then benefit from a positive psychological intervention if their symptoms are being addressed by another treatment. This should be explored in further research.

Finally, moving to the wider implications of the study, it has previously been argued that taking positive psychology online has the potential to address treatment needs through a resource that once developed does not require further therapist time or resources to deliver [6]. However, as mentioned, our findings indicate online positive psychology might be more engaging when used alongside other treatments or when supported by a therapist. Indeed, support has been identified as potentially critical in promoting adherence in other online interventions [11, 30]. Future research must establish the level and type of support that might be required, which is subject to debate in current literature [31, 32]. However, if the aim is for patients to use positive psychology as self-help, our findings indicate a possible solution is to explore how persuasive design could be leveraged to promote a sense of support and connectedness in order to promote adherence [33].

## Conclusions

The present study produced novel insights into the factors that could affect the acceptability of an online positive psychology intervention for people with depression and anxiety. The findings are likely to be valuable for generating ideas on how to design future online positive psychology interventions that are engaging and appealing, which can be developed in future research.

## Additional file

Additional file 1: Topic guide. This is the topic guide used in the semi-structured interviews, that includes key questions and suggested probes. (DOCX 60 kb)

## Abbrevaitions

Apps: Smartphone applications
COREQ: Consolidated criteria for reporting qualitative research
MRC: Medical Research Council
NHS: National Health Service
UK: United Kingdom
