# Supplementary material for: What might affect acceptability of online positive psychology interventions for depression: a qualitative study on patient expectations’
Source: BMC Psychiatry. 2018 Jul 27;18:240. doi: 10.1186/s12888-018-1812-x (PMC6062878; doi:10.1186/s12888-018-1812-x)
Supplement: Supplementary file 1 — Topic guide. This is the topic guide used in the semi-structured interviews, that includes key questions and suggested probes. (DOCX 60 kb) [file 12888_2018_1812_MOESM1_ESM.docx]

**Topic Guide: Developing an online positive psychological intervention for depression and anxiety**

**Introduction (5 minutes)**

- **Nature and focus of research**: the interview is about discussing their views on the approach of positive psychotherapy, i.e. interventions or treatments which focus on promoting wellbeing, happiness and satisfaction, rather than fixing problems or symptoms and general views on using technology to help support people living with depression and anxiety
- **Confidentiality**: The name of the participants will only be known by the researchers and not be revealed to anyone The participants will be identified by an ID and all potentially identifying information will be removed
- **Participation:** don’t share information you don’t want to, we don’t have to agree but all conversations must remain confidential
- **Recording:** the session will be audio-recorded, transcribed and analysed by researchers using NVIVO software, one of the most used widely software for qualitative analysis
- **Any questions…**…..

1. **General views on positive psychological approach (20 minutes)**

**Brief description mention focus on promoting 1) positive emotions (in the here and now, the past and the future), 2) engagement (described as awareness and use of in daily life) and 3) meaning (sense of connection with others in your life and your wider community)**

- Based on that initial description what would be your response if you were offered that treatment approach for your anxiety/ depression?
- Advantages of the positive approach if you were anxious/ depressed?
- Disadvantages of the positive if you were anxious/ depressed?
- What kind of help were you given when you were anxious/ depressed?
- What kind of help would you have liked when you were anxious / depressed?
- What was your preferred mode of delivery (e.g. self-help, computerised help, app, in person therapy) when you were anxious / depressed?
  - - Why that approach preferred?
- In terms of self-help then, for example as an app, would you have wanted it when you were anxious/ depressed?
  - Is there a time when it would have been suitable?
    - Waiting list
    - During therapy
    - After therapy

1. **Specific positive psychological exercsies** (20 minutes)

Outline each homework exercise from Positive Psychotherapy (detailed in Table 1) and ask

- How would you feel if you were asked to do this activity when you were feeling low in mood (depressed) or particularly worried (anxious)?
- How would you feel about completing this activity as self-help
- How would you feel about using technology, such as an app or website to do this activity?
- What might help you to benefit from such an activity?

1. **Personal online use for health (5 minutes)**

- You’ve mentioned that your use of website or apps to manage health in general is [insert answer to questionnaire] can you say a bit about why that is?
  - Thought of it?
  - Recommended any?
  - Tried any?

1. **Online structure and features (15 minutes)**

- How would you want to be introduced to this?
  - From who?
  - What kind of information?
- How important would it be to connect with other people through an intervention like this?
  - With who (e.g. GP, therapist, other users)
    - Advantages / disadvantages
- How should the intervention link with the NHS?
  - Branding
  - Links
- How should the intervention information be released, should all the information be presented at once or in stages?
- How often would you think you should use the intervention?

1. ***Design and branding (10 minutes)***
   - *What would be important to make the content appealing? (e.g. use of colour, font size, style of language)*
   - *Are there any online interventions that you particularly like the branding or style of?*
   - *Is there any images or looks you think the online intervention should avoid?*
2. **Closing interview (2 minutes)**
   - Anything important I need to think of that we haven’t touched on?
   - Re-affirm use of findings/confidentiality and feedback arrangements. Thank participants for their contribution
